# Thawed Mesenchymal Stem Cell Product Shows Comparable Immunomodulatory Potency to Cultured Cells *In Vitro* and in Polymicrobial Septic Animals

**DOI:** 10.1038/s41598-019-54462-x

**Published:** 2019-12-02

**Authors:** Yuan Tan, Mahmoud Salkhordeh, Jia-Pey Wang, Andrea McRae, Luciana Souza-Moreira, Lauralyn McIntyre, Duncan J. Stewart, Shirley H. J. Mei

**Affiliations:** 1 Regenerative Medicine Program, Ottawa Hospital Research Institute, Ottawa, Ontario K1H 8L6 Canada; 2 Clinical Epidemiology Program, Ottawa Hospital Research Institute, Ottawa, Ontario K1H 8L6 Canada; 3 Faculty of Medicine, University of Ottawa, Ottawa, Ontario K1H 8M5 Canada

**Keywords:** Mesenchymal stem cells, Stem-cell biotechnology, Regenerative medicine

## Abstract

Mesenchymal stem cells (MSCs) have been shown to exert immunomodulatory effects in both acute and chronic diseases. In acute inflammatory conditions like sepsis, cell therapy must be administered within hours of diagnosis, requiring “off-the-shelf” cryopreserved allogeneic cell products. However, their immunomodulatory potency, particularly in abilities to modulate innate immune cells, has not been well documented. Herein we compared the stabilities and functionalities of cultured versus thawed, donor-matched MSCs in modulating immune responses *in vitro* and *in vivo*. Cultured and thawed MSCs exhibited similar surface marker profiles and viabilities at 0 hr; however, thawed MSCs exhibited higher levels of apoptotic cells beyond 4 hrs. *In vitro* potency assays showed no significant difference between the abilities of both MSCs (donor-matched) to suppress proliferation of activated T cells, enhance phagocytosis of monocytes, and restore endothelial permeability after injury. Most importantly, in animals with polymicrobial sepsis, both MSCs significantly improved the phagocytic ability of peritoneal lavage cells, and reduced plasma levels of lactate and selected inflammatory cytokines without significant difference between groups. These results show comparable *in vitro* and *in vivo* immunomodulatory efficacy of thawed and fresh MSC products, providing further evidence for the utility of a cryopreserved MSC product for acute inflammatory diseases.

## Introduction

Mesenchymal stem cells (MSCs) have been shown to exert important immunomodulatory effects in both acute and chronic diseases. These effects include the modification of the inflammatory cascade and improvement of tissue repair and pathogen clearance, all of which are central to immune-mediated diseases such as sepsis or acute respiratory distress syndrome (ARDS)^1–5^. We and others have demonstrated beneficial effects of MSCs in the clinically relevant cecal ligation and puncture (CLP) model of polymicrobial sepsis, in which MSCs promoted host immune response related to bacterial clearance, reduced markers of organ failure and most importantly, improved animal survival^3,4^. As MSCs lack histocompatibility complex class II amongst other immune-active co-stimulatory molecules^6^, these cells are believed to be immune-privileged and can be used in allogeneic fashion. To treat acute inflammatory conditions under clinical settings, a cryopreserved, allogeneic MSC product available for delivery within hours of diagnosis is a more logistically and economically feasible therapy compared to a product using freshly cultured cells. While “off-the-shelf”, cryopreserved allogeneic cell products have been used in a number of clinical trials, including for acute inflammatory diseases such as ARDS^7^, preclinical results supporting their potency compared to freshly cultured MSCs are conflicting.

Early preclinical studies suggest that cryopreserved MSCs may have reduced immunomodulatory activities immediately post-thaw, thus may not be as effective as MSCs harvested immediately from culture. Some studies have shown that the *in vitro* abilities of MSCs to inhibit proliferation of activated T cells, respond to pro-inflammatory stimuli, and produce anti-inflammatory mediators were impaired in thawed MSCs compared to cultured MSCs^8,9^. Moreover, thawed MSCs have been reported to trigger an innate immune response (instant blood mediated inflammatory reaction; IBMIR) and activate the complement cascade compared to cultured cells, raising concern for therapeutic use of thawed MSCs in patients^10^. In contrast, several recent publications suggest that a cryopreserved-then-thawed MSC product have similar or even superior therapeutic potency to a freshly cultured MSC product^11–14^. Given the contrasting results reported to date, whether a cryopreserved-then-thawed MSC product could serve as a comparably effective therapeutic substitute for freshly cultured cells is still debatable, especially for acute inflammatory conditions like ARDS or sepsis.

To specifically address this question, we compared the immunomodulatory potential of cultured versus thawed MSCs using several *in vitro* potency assays, as well as in an animal model of acute inflammatory disease (i.e., sepsis). Cultured and thawed, donor-matched MSCs, manufactured with xeno-free, GMP-grade culture media, were evaluated for MSC phenotype, including viability and surface marker expression. Immunomodulatory functions of MSCs were assessed by their ability to suppress proliferation of activated T cells, enhance phagocytotic capacity of monocytes and reduce permeability of an endothelial cell (EC) monolayer. Finally, the ability of the cultured and thawed MSCs to reduce inflammation and improve pathogen clearance was assessed in a CLP murine model of polymicrobial sepsis.

## Results

### Thawed MSCs show comparable surface marker profile, viability and recovery to cultured MSCs in short-term stability study

**Figure 1:**
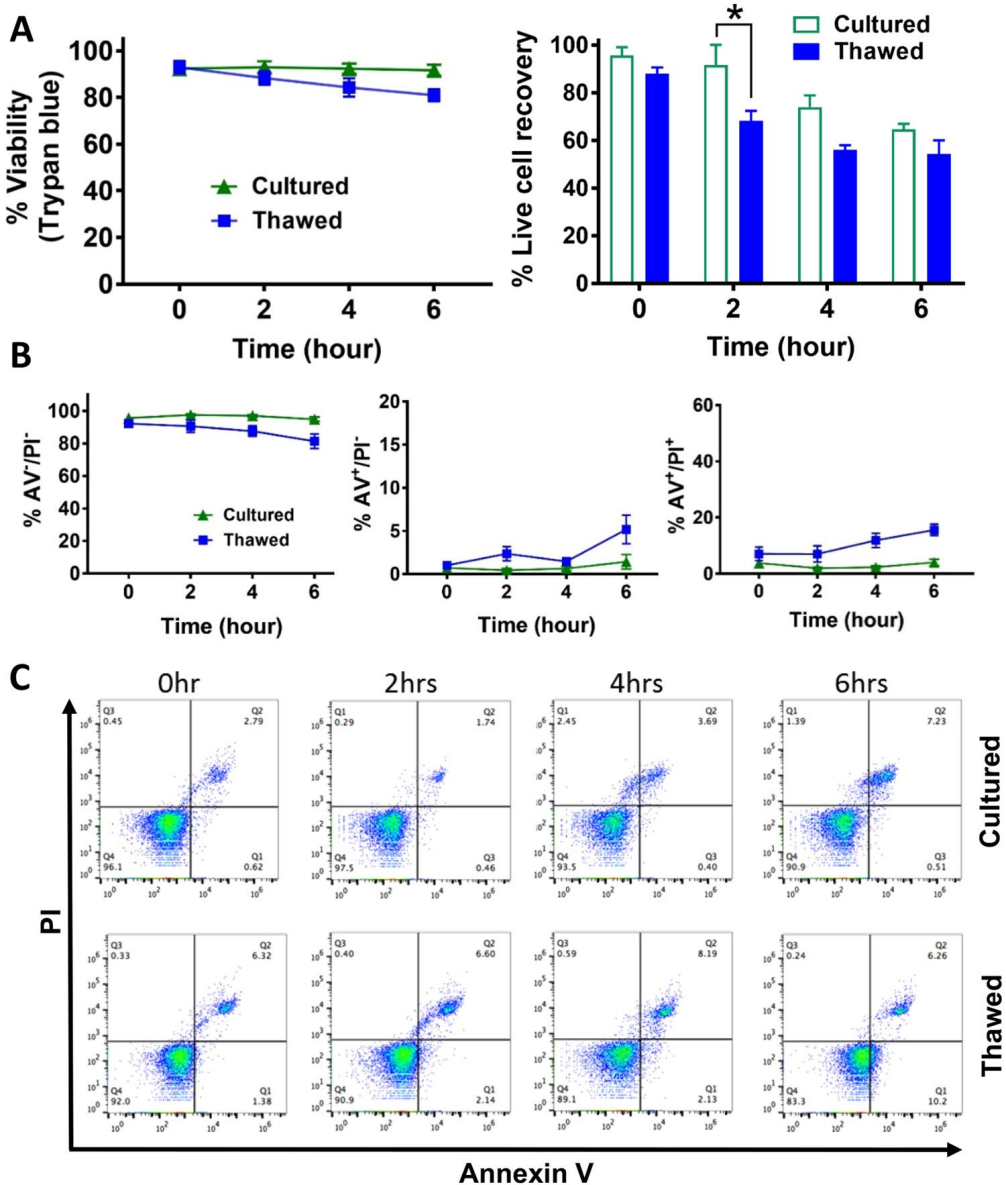
Assessment and comparison of cell viability of cultured and thawed MSCs over 6 hours. (**A**) Trypan blue exclusion method was used to assess viability (left panel) and calculate viable cell recoveries (right panel), with measurements taken at 0, 2, 4, and 6 hours. (**B**) Annexin V (AV) and Propidium Iodide (PI) staining of cells followed by flow cytometry analysis was used to assess populations of cells that were viable (left panel) and going through early (middle panel) or late (right panel) apoptosis, with measurements taken at 0, 2, 4, and 6 hours. (**C**) Representative flow cytometry data of AV/PI stained cells demonstrating percentage of late apoptotic cells (AV+/PI+, Q2); early apoptotic cells (AV+, Q3), and live cells (AV−/PI−, Q4). *n* = 3 independent experiments, with data represent mean ± SEM.

To determine whether thawed MSCs performed similarly to cultured MSCs within the first 6 hours after preparation, cell viability and cell recovery were compared. Viability was measured with Trypan blue exclusion at 0, 2, 4, and 6 hours post-thaw (i.e., thawed MSCs) or harvest (i.e., cultured MSCs). While there were no significant differences in cell viability for cultured and thawed MSCs at 0 hours (92% ± 2.7% and 93% ± 2.6%) or 6 hours (91% ± 2.3% and 81% ± 2.5%, respectively) (Fig. 1A), as expected this was slightly lower for the thawed cell product at the later time point. Again, cell recovery was slightly lower for thawed vs. fresh cells, but this was only significant at 2 hours (*p* < 0.05) after preparation. A similar trend in cell viability was observed in AV/PI staining results, with a slightly lower proportion of viable cells at 6 hours for thawed MSCs (Fig. 1B,C; p < 0.05), which was associated with increases in both early apoptotic (AV^+^/PI^−^) and late apoptotic cells (AV^+^/PI^+^), although this was only significant at 4 hours for the apoptotic population; and 6 hours for live, early, and late apoptotic populations (*p* < 0.05).

**Figure 2:**
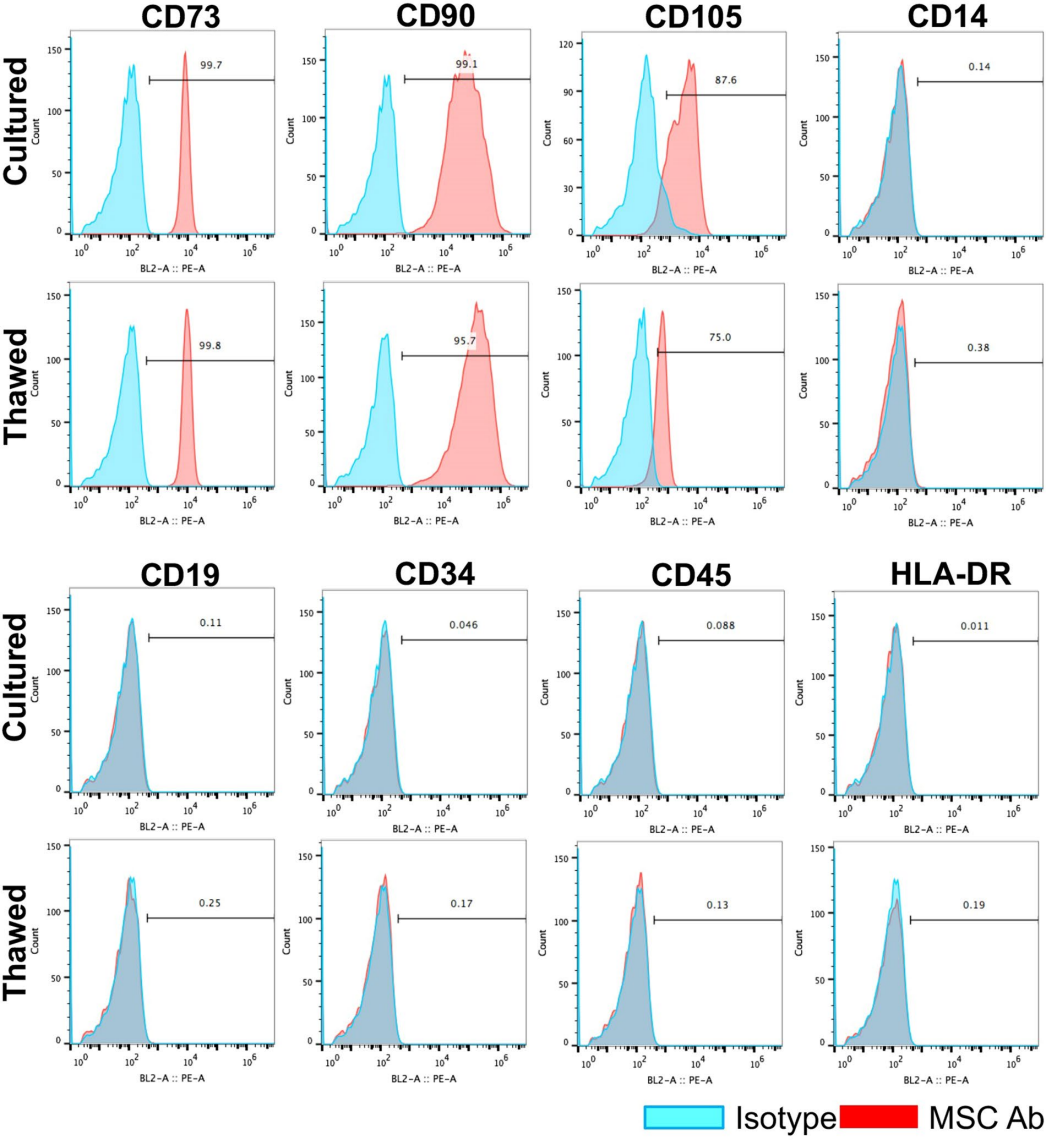
Surface marker profiles for cultured and thawed MSCs. Representative flow cytometric plots indicate positive markers: CD73, CD90, CD105; negative markers: CD14, CD19, CD34, CD45, and HLA-DR.

To compare and confirm MSC identity for cultured and thawed cells, surface marker profiling experiments by flow cytometry analysis were performed at 4 hours post-thaw or harvest (Supplementary Table S1), with representative flow cytometric plots shown in Fig. 2. No difference was observed between the cultured and thawed MSCs in surface marker expression (characterized by positive markers CD73, CD90, and CD105, and negative markers CD14, CD19, CD34, CD45, and HLA-DR).

### Thawed MSCs show comparable *in vitro* potency to cultured MSCs

**Figure 3:**
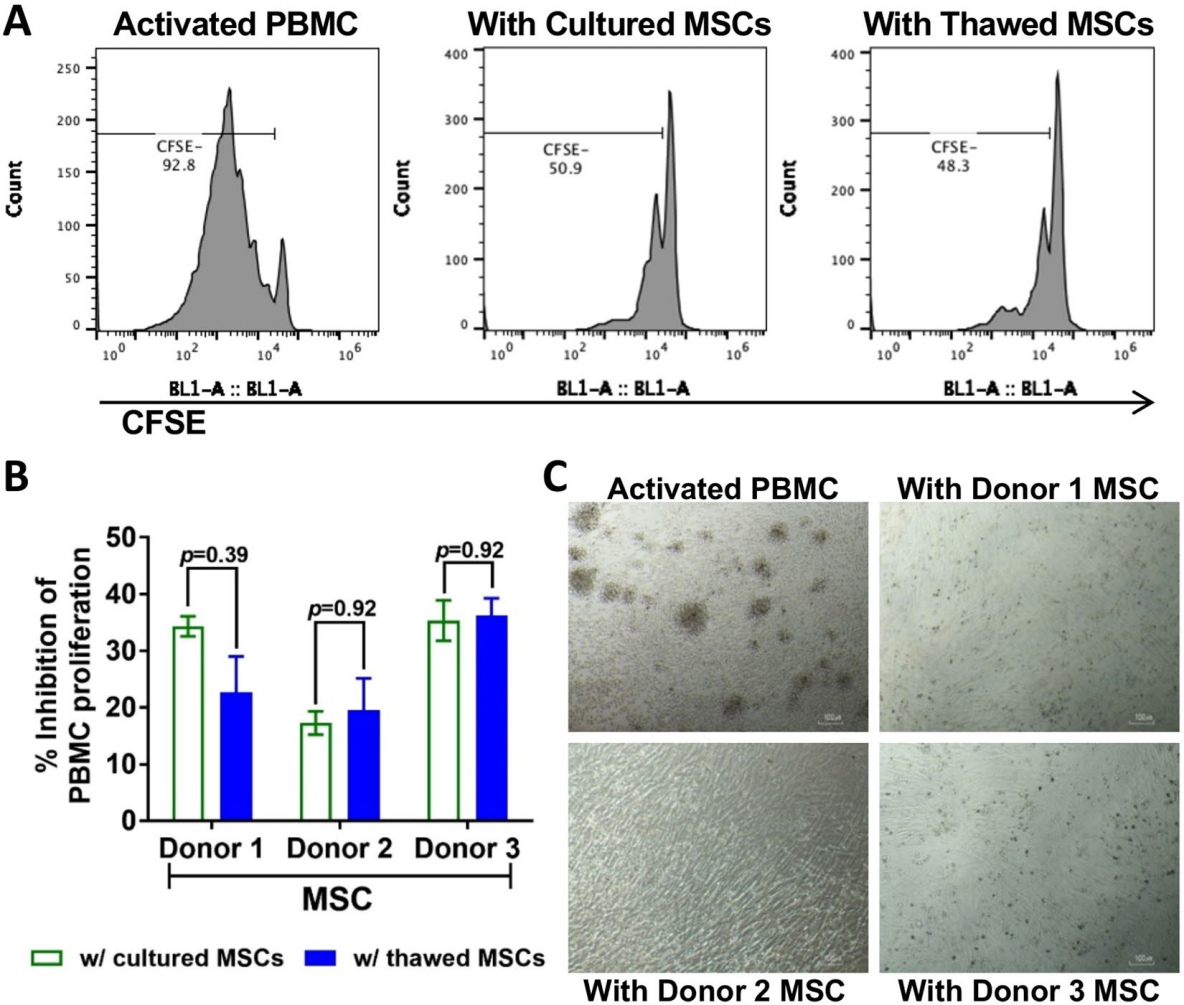
Cultured and thawed MSCs were comparably potent in inhibiting T cell proliferation. PBMCs were stained with carboxyfluorescein succinimidyl ester dye (CFSE), then activated with anti-CD3/CD28 Dyna-beads. CFSE-labeled PBMCs were co-cultured with MSCs for 5 days before analysis by flow cytometer. (**A**) Representative CFSE histograms of activated PBMCs without MSCs, activated PBMCs co-cultured with cultured MSCs, or activated PBMCs co-cultured with thawed MSCs. (**B**) Percent inhibition of activated PBMCs after co-culture with cultured or thawed MSCs. *n* = 3~6 experiments, with bars represent mean ± SEM (*2-tailed unpaired t-test corrected with Holm-Sidak method*). (**C**) Microscopic images of activated PBMC without MSCs (shown as aggregated colonies), or with different donor-derived MSCs after co-culture for 5 days. Scale bar = 100 µm.

Thawed and cultured MSCs were compared for their *in vitro* potency using three assays. One evaluated the ability of MSCs to modulate adaptive immune cell response, two other assays were developed to assess the ability of MSCs to enhance monocyte phagocytic activity and restore impaired EC permeability. To examine the extent of MSCs capacity to suppress proliferation of peripheral blood mononuclear cells (PBMCs), CD3/CD28-activated, CFSE-labeled PBMCs were analyzed by a flow cytometer and showed 92.8% proliferation after 5 days of culture (Fig. 3A). Co-culturing of activated PBMCs with cultured or thawed MSCs reduced numbers of proliferating PBMCs to 56.8% and 44.3% on representative flow cytometric plots, respectively (Fig. 3A). Although donor-to-donor variability was apparent, on average ranging from 13% to 38% inhibition of T cell proliferation (Fig. 3B), both cultured and thawed MSCs from any given donor had equivalent inhibitory activity on PBMC proliferation. Figure 3C shows that MSCs reduced number of aggregated colonies as further evidence of inhibitory activity.

**Figure 4:**
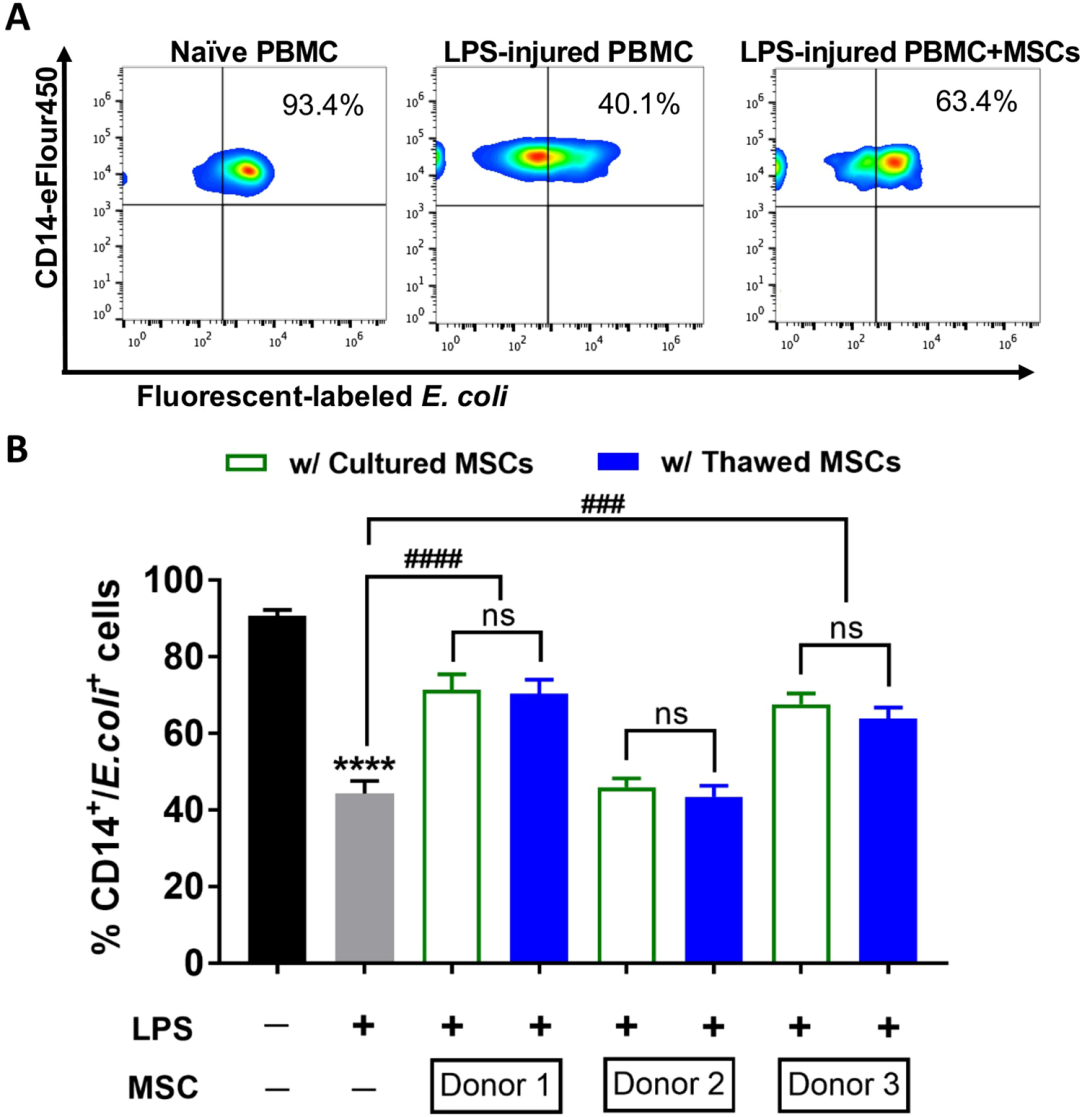
Effect of cultured and thawed MSCs on PBMC’s phagocytic capacity. (**A**) Representative flow cytometric plots of naïve PBMCs, LPS-treated PBMCs without and with MSCs demonstrating the PBMC’s ability to phagocytose bacteria as indicated by the percentage of CD14+ cells positive for green fluorescent signal. (**B**) Naïve PBMCs were pre-treated with LPS for 18 hours and co-cultured with cultured and thawed MSCs from three different donors for 24 hours. PBMCs were harvested and incubated with fluorescent tagged *E. coli* and analysed by flow cytometry to show the percentage of CD14+/*E. coli* positive cells. *n* = 3~6 experiments, with bars representing mean ± SEM. Group comparisons were analyzed by one-way ANOVA with Bonferroni’s post hoc test. ****p < 0.0001, LPS-treated PBMC versus non-treated group. ns = not significant between groups compared.

**Figure 5:**
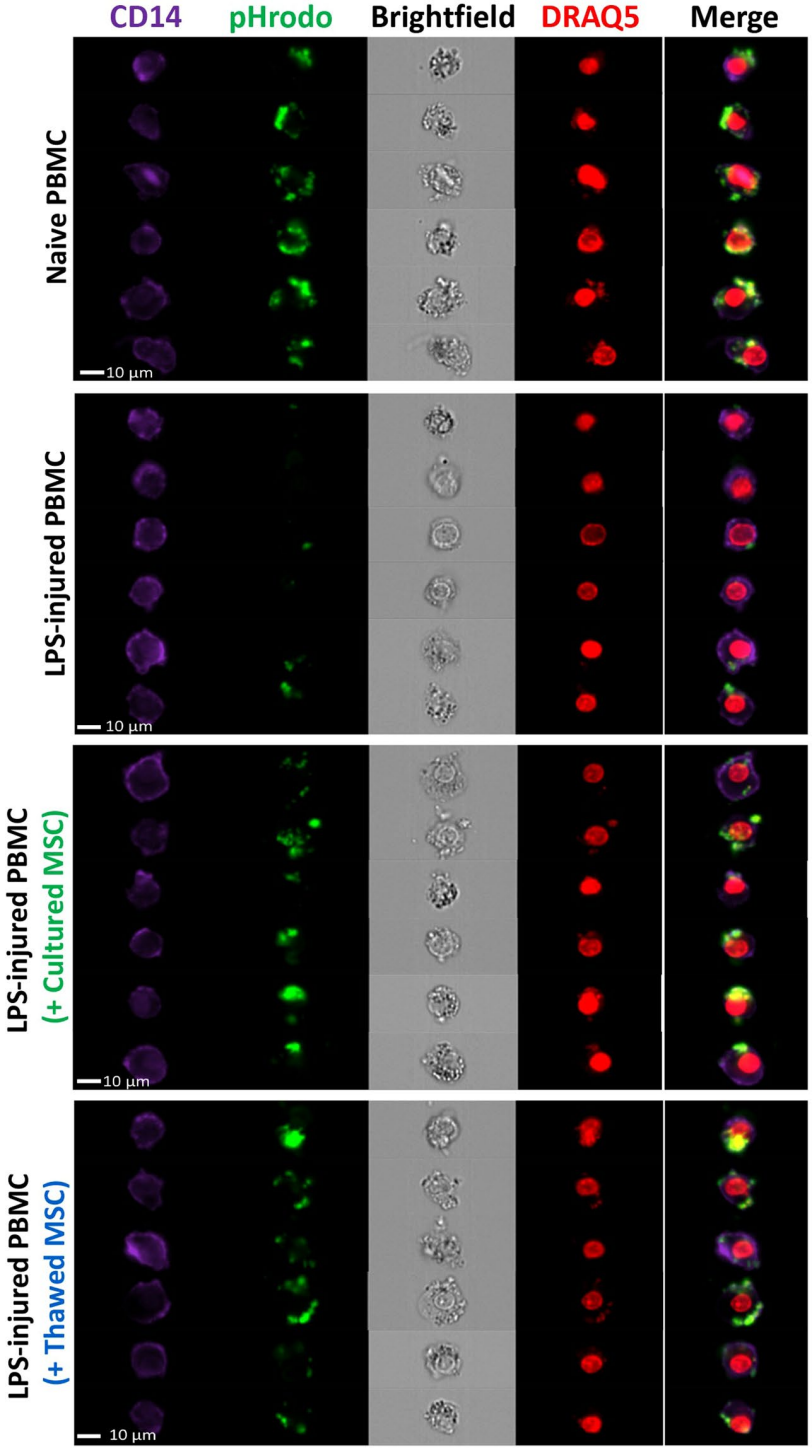
Single cell imaging of PBMC’s capacity to engulf fluorescent bacteria. AMNIS Imaging flow cytometer digital images show representative CD14+ PBMC populations (purple) and the ability of cultured and thawed MSCs to rescue the PBMC’s capacity to engulf the pHrodo Bioparticles (green). The PBMC nuclei are stained with DRAQ5 in red. The morphology of the cells is shown by bright-field microscopy and the PBMC nuclei are stained with DRAQ5 in red. Scale bar = 10 µm.

An *in vitro* phagocytosis assay was conducted to examine the ability of cultured and thawed MSCs to enhance bacterial phagocytosis by CD14+ PBMCs. Our group previously demonstrated that MSCs possess the ability to modulate host response to bacterial infection^3^, potentially through up-regulation of genes responsible for promoting phagocytosis and killing of bacteria^1,3^. Naïve CD14+ PBMC cells exhibited a high level of phagocytosis on average, with 91% ± 1.5% of CD14+ cells being positive for fluorescently tagged *E. coli*; whereas LPS treatment caused marked reduction of phagocytic capacity on CD14+ PBMCs, with only 44% ± 3.3% of CD14+ cells with positive signals (*p* < 0.0001 compared to naïve CD14+ CD14+ PBMCs) (Fig. 4A). After co-culturing with MSCs for 24 hours, the ability of CD14+ cells to phagocytose *E. coli* was partially recovered (*p* < 0.0001 for Donor 1, and *p* < 0.001 for Donor 3). Thawed MSCs from three different donors showed comparable improvement in potency to the donor-corresponding cultured MSCs (no significant difference treated with either cultured or thawed MSCs within a given donor), ranging from 71% ± 4.1% (Donor 1) to 46% ± 2.4% (Donor 2) percent of CD14+/*E. coli*+ positive population, respectively (Fig. 4B). The CD14+ cell population engulfing the fluorescent bacteria in the presence or absence of MSC groups can be visualized by single-cell flow cytometry-based imaging as seen in Fig. 5.

**Figure 6:**
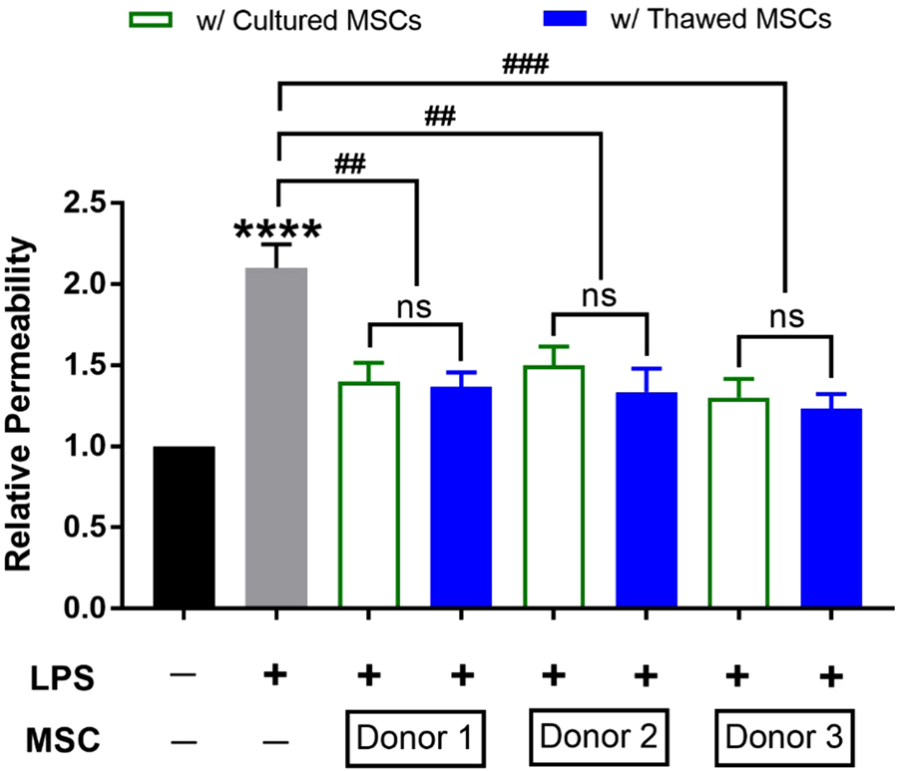
Cultured and thawed MSCs were comparable in their abilities to recover endothelial cell permeability post-LPS injury. Endothelial cells (ECs) were seeded in a transwell and treated with LPS for 6 hours. Cells were then co-cultured for 24 hours with MSCs that were seeded in the lower receiver wells. After co-culture, FITC-dextran was added to the top of the transwells. Permeability of the EC monolayer was determined by taking samples from the lower compartment to measure FITC-dextran levels. Group comparisons were analyzed by one-way ANOVA with Bonferroni’s post hoc test. ****p < 0.0001, LPS-treated ECs versus non-treated group. ns = not significant between groups compared.

Endothelial damage or dysfunction, specifically the increase of vascular permeability and loss of endothelial integrity, can contribute to the pathophysiological progression of sepsis^15^. To assess the ability of MSCs to restore barrier function of an endothelial monolayer after injury, permeability to FITC-labelled dextran was assessed in an *in vitro* transwell assay. The presence of LPS-induced endothelial injury was shown by an increase in relative permeability by 2-fold (*p* < 0.0001). Co-culture with either cultured or thawed MSCs from three different donors significantly decreased EC permeability, compared to the LPS-treated control (*p* < 0.01 for Donors 1 and 2; *p* < 0.001 for Donor 3), with no significant difference between ECs co-cultured with either cultured or thawed donor-specific MSCs (Fig. 6). Together these data suggest that the potency of cultured and thawed MSCs was similar in terms of immunomodulatory and endothelial cell protective effects *in vitro*.

### Cultured and Thawed MSCs equally improve bacterial clearance and levels of systemic inflammation in a CLP model

**Figure 7:**
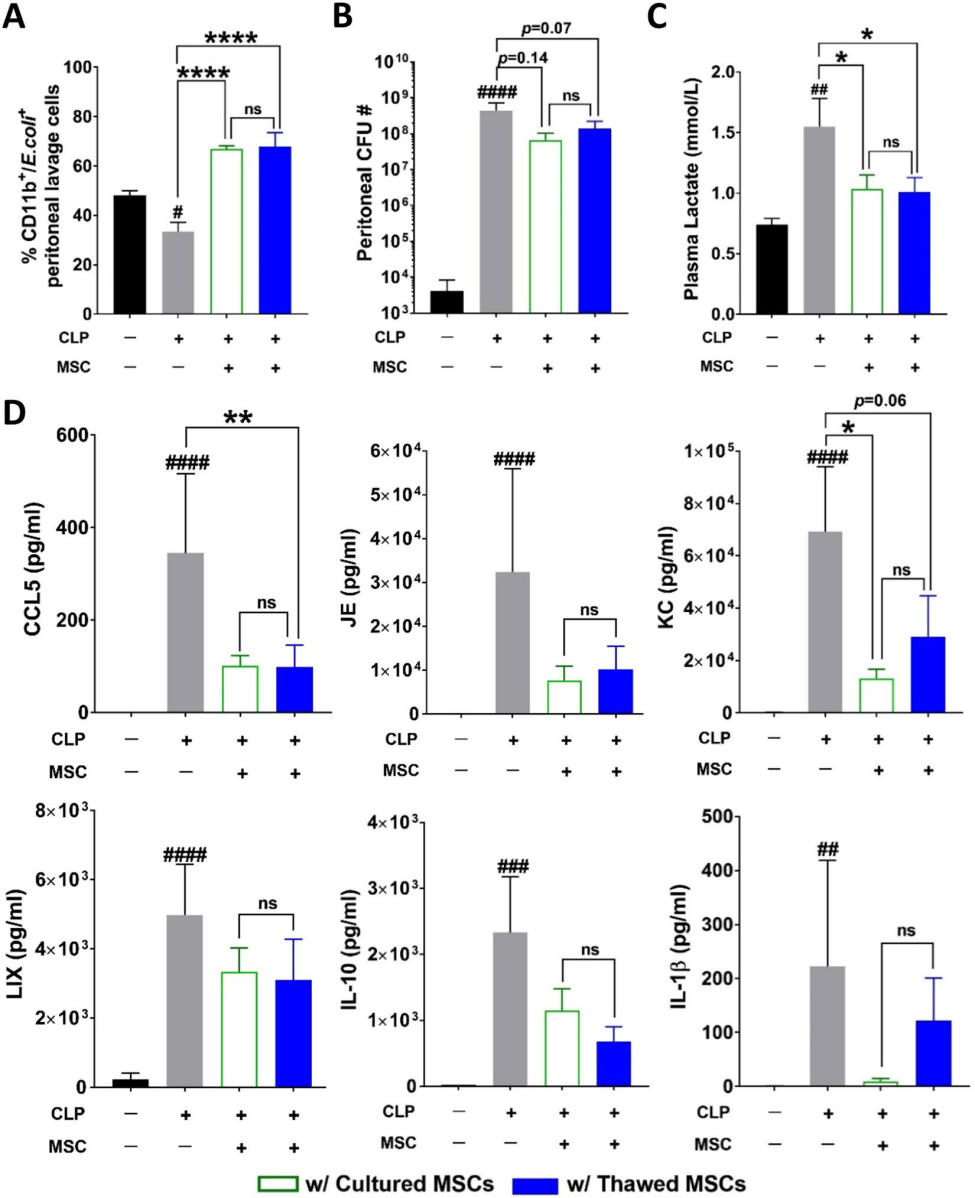
Cultured and thawed MSCs were comparable in improving bacterial clearance and systemic inflammation in a murine model of acute inflammatory injury (cecal-ligation-puncture model). (**A**) Peritoneal lavage cells were incubated with fluorescent tagged *E. coli* followed by flow cytometric analysis to assess the ability of the peritoneal cells to phagocytose bacteria. (**B**) Peritoneal lavage fluid was plated on blood agar to quantify the colony forming units (CFUs) and determine the peritoneal bacterial load. Plasma samples were evaluated to detect (**C**) lactate, and (**D**) cytokine levels of systemic inflammation. Group comparisons were analyzed by one-way ANOVA with Dunnett’s post hoc test. ^#^*p* < 0.05, ^###^*p* < 0.001 and ^####^*p* < 0.0001, sham/vehicle versus CLP/vehicle group. *p < 0.05, **p < 0.01 and ****p < 0.0001, CLP/saline versus MSCs-treated group. ns = not significant between groups compared.

To investigate the ability of the MSC treatment groups to modulate immune responses, the *in vivo* potency of the MSCs was assessed by their ability to improve bacterial clearance and modulate cytokine levels in an animal model of acute inflammatory injury (i.e., cecal-ligation-and-puncture; CLP). CD11b+ cells from peritoneal lavage of CLP mice exhibited a marked reduction in their ability to phagocytose live bacteria, compared to cells from sham-operated mice (*p* < 0.05, Fig. 7A). Intravenous administration of either cultured or thawed MSCs significantly ameliorated the phagocytic ability of the peritoneal lavage cells with a 2-fold increase in cells positive for *E. coli* (*p* < 0.0001 for cultured and thawed MSC groups compared to CLP/vehicle control). Next, we examined the ability of MSC treatment groups to clear local bacterial growth in septic mice. As expected, bacterial CFU counts were high in the peritoneal lavage fluid of mice that had undergone CLP compared to sham-operated animals (*p* < 0.001) (Fig. 7B). Treatment with cultured or thawed MSCs reduced CFU counts 24 hours post-CLP (*p* = 0.14 for CLP mice treated with cultured MSCs or *p* = 0.07 for CLP mice treated with thawed MSCs, compared to CLP/vehicle control) (Fig. 7B). No significant difference in peritoneal CFUs was apparent between MSC treatment groups.

High lactate level is typically seen in sepsis; therefore, it was measured from plasma samples collected at the end of study. Plasma lactate levels were significantly elevated in the CLP/vehicle control group compared to the sham-operated mice (*p* < 0.01) (Fig. 7C). Administration of either cultured or thawed MSCs markedly reduced plasma lactate levels (*p* < 0.05 for cultured or thawed MSC groups, compared to CLP/vehicle control) and no significant difference between MSC treatment groups was evident (Fig. 7C). Compared with sham-operated mice, plasma concentrations of chemokines CCL5 (murine homolog of human RANTES), JE (murine homolog of MCP-1), KC (murine homolog of IL-8), and LIX (murine homolog of ENA-78) were markedly elevated in the CLP/vehicle control group (*p* < 0.0001 for CCL5, JE, KC, and LIX) (Fig. 7D). Plasma levels of the proinflammatory cytokine IL-1β and the anti-inflammatory cytokine IL-10 were also elevated in mice with CLP-induced sepsis compared to sham-operated mice (*p* < 0.001 and *p* < 0.01 for IL-1β and IL-10, respectively) (Fig. 7D). Treatment with cultured MSCs reduced all systemic cytokine and chemokine (IL-10, IL-1β, JE, CCL5, LIX, and KC) levels measured, in some instances (i.e. IL-1β) approaching levels observed in sham-operated mice. Treatment with thawed MSCs significantly reduced CCL5 levels (*p* < 0.01) with trends towards reduced levels of JE, KC (*p* = 0.06), LIX, IL-10, and IL-1β (Fig. 7D). No significant difference in the cytokine and chemokine levels measured was apparent between the cultured and thawed MSC treatment groups.

## Discussion

In the present study, we demonstrated that cultured and thawed MSCs exhibited the same surface marker profiles and similar cell viabilities immediately after preparation, with thawed MSCs only starting to show increased levels of early and late apoptotic cells beyond 4 hours. *In vitro* assays highlighted no significant difference between cultured and thawed MSCs (donor-matched and manufactured with xeno-free, GMP-grade culture media) in their abilities to suppress proliferation of activated T cells, enhance phagocytosis of monocytes, and restore endothelial permeability after injury. More importantly, in our animal model of sepsis study, both cultured and thawed MSC treatments significantly improved the phagocytic ability of the peritoneal lavage cells, and reduced plasma levels of lactate and selected inflammatory cytokines, compared to untreated septic animals. Notably, there was no significant difference observed between the aforementioned effects seen using either cultured or thawed MSCs. These results demonstrate that thawed and cultured MSC products have comparable cell characteristics and *in vitro* and *in vivo* immunomodulatory efficacy.

Previous preclinical reports suggest that cryopreserved and thawed MSCs exhibit reduced immunomodulatory capacity^8,9^, and that thawed MSCs could potentially trigger an innate immune attack by the host via activation of complement cascade, causing lysis of MSCs and resulting in impaired therapeutic efficacy^10^. In contrast, more recent publications indicate that cryopreserved MSCs administered immediately after thawing may have comparable or equivalent therapeutic potency to freshly cultured MSCs. Leutzendorf *et al*. used co-culture assays of MSCs with PBMCs and demonstrated that cryopreservation and immediate thaw of MSCs had no impact on immunogenicity and immunomodulatory capacity of the MSCs^11^. This was further supported by Gramlich *et al*., who showed that thawed and cultured MSCs *in vitro* had no significant difference in metabolic activity, growth factor secretion, and immunomodulatory potency, such as the ability to inhibit T cell proliferation and an increased enzyme indoleamine-pyrrole 2,3-dioxygenase (IDO) expression after IFNγ priming^12^. These cells were subsequently administered into animals with retinal ischemia/reperfusion injury as a model of central nervous system injury. Both thawed and cultured MSC therapy rescued the retinal ganglion cells, which was a measure of therapeutic efficacy. In another study, an immunocompetent mouse model of allergic airways inflammation was used with no difference in effects of cultured versus thawed human MSCs (hMSCs) apparent on lung mechanics and inflammation, although some variability in the effects on bronchoalveolar lavage fluid composition was noted^13^. Even in a rodent *E. coli* model of acute lung injury (ALI) using immunocompetent rats, hMSCs maintained efficacy after cryopreservation as well as decreasing *E. coli* bacteria counts, and reducing severity of lung injury^14^. This may explain the recent interest in other modes of action by MSCs, with a novel notion that the immunomodulatory activity of MSCs may involve phagocytosis of MSCs by macrophages^16^, or extracellular vesicle-mediated mitochondrial transfer to polarize macrophages to an immunosuppressive phenotype^17^. Interestingly, a study by Galleu *et al*. showed that MSC apoptosis may be necessary for their *in vivo* immunomodulation in an animal model of graft versus host disease (GvHD)^18^.

The finding that thawed MSCs were as potent, if not even more so, than cultured MSCs in improving phagocytosis capacity of peritoneal CD11b+ cells and suppressing various inflammatory circulating cytokines *in vivo* is consistent with our *in vitro* data and similar to a study conducted by Devaney *et al*.^14^. Using three different donor-derived MSCs, we have demonstrated that thawed MSCs retained their therapeutic potency as well as the cultured cells in three *in vitro* assays, with thawed MSCs being on average 91% as potent as the cultured cells in inhibition of T cell proliferation, 96% as potent in improving phagocytosis after LPS injury, and 94% as potent in rescuing LPS-induced endothelial damage. Furthermore, the similar abilities of cultured and thawed MSCs for the enhancement of monocyte phagocytosis seen using an *in vitro* assay was further verified *in vivo* for the first time by using a murine model of polymicrobial sepsis with the CLP model. Overall, our study provides new evidence on the comparability of cultured and thawed MSC products, specifically examining the immunomodulatory effects of MSCs in acute inflammation and innate immunity, while most previous work in this field almost exclusively focused on studying the effect (or lack thereof) of thawed MSCs on the modulation of T cell-related activities.

While the majority of cell therapy studies have utilized cultured MSC products in animal models and even small clinical trials, a cryopreserved cell product may be essential to enable a broader application and distribution of MSC-based therapies in human patients, particularly for conditions that are acute in nature (i.e., sepsis or ARDS). Cryopreservation and storage are a readily routine process for cells, tissues, and engineered cell therapy products^19,20^ and can be stable for weeks at −80 °C for off-the-shelf uses, thus eliminating continuous cell culture on-site. Studies investigating the impact of cryopreservation on MSC function have yielded mixed results. In particular, how cell viability would contribute to therapeutic benefit remains an important issue. After Moll *et al*. first suggested the concept of efferocytosis as a means by which apoptotic MSCs may drive immunosuppression^21^, contradictory evidence has also emerged to indicate that the potency of apoptotic MSCs might be substantially less than that of live MSCs. Nevertheless, these are just some examples that illustrate the complicated mode of action a cellular therapeutic may employ compared to chemical-based pharmaceuticals^16^.

It is noteworthy that, in many studies showing inferior potency results with thawed MSCs, poorer viabilities have typically been reported and implicated in the therapeutic efficacy of cells. In a recently published Phase 2 clinical trial (START study: Treatment with allogeneic mesenchymal stromal cells for moderate to severe ARDS), Matthay *et al*. found no difference between patients treated with or without MSCs, primarily on 28-day patient mortality rates. However, the cryopreserved allogeneic MSCs exhibited variable viability from as low as 36% to as high as 85%^22^, which may in part contribute to the lack of overall efficacy seen in the treatment group. The authors proposed that the extra cell wash procedure, which was implemented to remove the cryopreservant agent dimethyl sulfoxide (DMSO), may account for the wide range of viabilities seen in their MSC product, as cells were vulnerable to the stress of washing and centrifugation procedures immediately after thaw. The authors further showed that simple thawing and dilution yielded notably greater MSC viability compared to the MSCs that had been washed, centrifuged, and reconstituted. In a preclinical study by Moll *et al*., where thawed cells were found to have inferior immunomodulatory properties and were more prone to trigger the host’s innate immune attack, the cryopreserved MSCs were thawed, washed twice, and then reconstituted before being used^10^. In contrast, Devaney *et al*. showed that cryopreservation of MSCs only modestly reduced cell viability, from cultured MSCs at 95.1% ± 0.6% to thawed MSCs at 91.8% ± 0.6%. Not only was there no loss in efficacy of cryopreserved MSCs to reduce lung injury severity, but furthermore, there was no added advantage to remove the cryopreservation solution prior to administration^14^. It is unclear whether the additional washing step implemented by some of these studies does in fact contribute to the loss of thawed MSC potency. Of note in our current study, as we do not employ a washing step to remove DMSO, the cell viability of our thawed MSCs were consistently over 90% after thawing, compared to that of 95% viability of cultured cells on average. To date, MSC therapies are being tested in more than 900 registered clinical trials^23,24^. Many trials have utilized a cryopreserved then thawed cell product, starting with the Osiris trial of allogeneic cryopreserved MSCs (Prochymal) for treating steroid-refractory GvHD in 2009. Though most of these are early-phase trials, there is growing evidence of efficacy with thawed MSC products, such as improving ventricular ejection fraction post myocardial infarction^25^ and promoting neuronal repair in secondary progressive multiple sclerosis^26^. Nevertheless, more research is needed to further optimize cryopreservation methods in addition to thawing/preparation processes for any given MSC product to advance the exciting field of cell therapy.

## Conclusion

This study addresses the question of whether a thawed MSC product compared to a cultured MSC product exhibits a similar stability profile and immunomodulatory potency using a range of *in vitro* assays and a polymicrobial sepsis animal model, and offers additional evidence for the utility of a cryopreserved, ready-to-be-thawed MSC product in patients with acute inflammatory conditions such as sepsis or septic shock. A cryopreserved-then-thawed MSC product with good viability and recovery requires careful and rigorous cryopreservation protocol development, which may involve testing multiple cryopreservation solutions to customize based on specific applications, accompanied by the utilization of an optimized and standardized freezing protocol. Furthermore, extra care must be taken to thaw and prepare the product before testing in different potency assays on the bench or for the administration at the bedside.

## Materials and Methods

### MSC culture

Bone marrow aspirates were obtained from the spinal fluid at the Intensive Care Unit, Ottawa Hospital, Ottawa, Canada with informed written consent and ethical approval granted by the Ottawa Health Science Network Research Ethics Board (REB ID: 20120929-01 H). All methods were performed in accordance with the relevant guidelines and regulations. Fresh, unprocessed human bone marrow (single donor) was also received from Lonza (Cat. 1M-105; Lot 0000348646; Lonza Walkersville, Inc.). Human bone marrow-derived MSCs were isolated, cryopreserved and cultured as described in the Supplemental Information. Cryopreserved MSCs that were thawed and cultured for at least 24 hours prior to *in vitro* and *in vivo* assays are termed “cultured MSCs”, while MSCs thawed immediately before testing are termed “thawed MSCs”. All experiments used cultured MSCs at passage 5, and thawed MSCs at passage 4. The capacity of these MSCs to differentiate into adipocytes, osteocytes, and chondrocytes was previously demonstrated (Supplementary Fig. S1). For *in vivo* studies, MSCs were suspended in a vehicle containing 5% human albumin (HA; Alburex 25, Canadian Blood Services) in PlasmaLyte A (PLA; pH 7.4, Baxter).

### Viability assessment

Cell viability was assessed using the Trypan blue exclusion method, or Annexin V (AV) (BD Biosciences) and propidium iodide (PI) (Thermo Fisher Scientific) flow cytometry analysis. Viable cell recoveries were calculated by dividing the total number of live cells counted post-thaw by the number of cells frozen down in the vial. For flow cytometry analysis, cells were stained with AV and PI for 15 minutes at room temperature according to manufacturer’s instructions. Flow cytometry (Attune Acoustic Focusing cytometer, Invitrogen) analysis determined the population of live, early, or late apoptotic MSCs.

### MSC surface marker profile

Immune-phenotype of MSCs from all donors was analyzed by flow cytometry detecting surface marker expression of CD73, CD90, and CD105; and lack of CD14, CD19, CD34, CD45, and HLA-DR (BD Biosciences) expression. MSCs were trypsinized, washed, and resuspended in cold PBS supplemented with 3% fetal bovine serum (FBS, Life Technologies) and stained with specific antibody against the aforementioned markers at 4 °C for 30 minutes according to manufacturer’s instructions. After washing, MSC surface markers were detected by flow cytometer and analyzed by FlowJo 10.0 software (FlowJo, LLC) using isotype control (PE-IgG1𝜿 and PE-IgG2a) (BD Biosciences) to set gating.

### *In vitro* potency assays

The inhibition of T cell proliferation assay used peripheral blood mononuclear cells (PBMCs) stained with carboxyfluorescein succinimidyl ester (CFSE) (Fisher Scientific) and activated with Dynabeads Human T-Activator CD3/CD28 (Gibco). The cultured MSCs were in culture for 24 hours prior to co-culture, while the thawed MSCs were thawed on the day of co-culture with PBMCs. Both cell groups were counted, seeded, and then co-cultured with the activated PBMCs at a ratio of 1:3 for 5 days then measured by flow cytometer.

The phagocytosis assay used PBMCs pre-treated with lipopolysaccharides (LPS; 100 ng/mL) first followed by co-culture with the MSC groups at a ratio of 1:5 for 24 hours. PBMCs were harvested and incubated with green fluorescent tagged *E. coli* Bioparticles (pHrodo; Invitrogen) and then stained with mouse anti-human CD14 conjugated with BV421 antibody (BD Biosciences) for dye-based detection of phagocytosis by Attune Acoustic Focusing cytometer (Invitrogen) and AMNIS ImageStream^X^ Mark II Imaging flow cytometer to capture single cell images (EMD Millipore Corporation) and analysis by IDEAS software (Millipore Sigma). Gradient Raw Mean Squared (RMS) for cells in focus was used for all events, and single cell events were gated using aspect ratio value and the area combining with DRAQ5 nuclear stain (Invitrogen).

The *in vitro* EC permeability assay seeded endothelial cells in a transwell insert (Corning) followed by LPS treatment for 6 hours. MSCs were then seeded in the lower receiver wells and co-cultured for 24 hours at ratio of 1:2 followed by adding FITC-dextran to the transwell insert (Sigma). The FITC-dextran mean fluorescence intensity level (MFI) of each group was measured with an aliquot taken from the lower compartment using the POLARstar omega fluorescence plate reader (BMG). Relative permeability was calculated as the MFI of treatment group divided by control group (untreated ECs).

### CLP model of sepsis

All animal experiments were conducted following ethical approval by the University of Ottawa Animal Care Committee (Ottawa, Canada) and complied with the principles and guidelines of the Canadian Council on Animal Care. Care of animals was in accordance with Canadian Council of Animal Care guidelines. All studies used 8-week old, healthy C57BL/6J female mice obtained from Charles River Laboratories (Kingston, ON). After receiving either CLP or sham procedure, animals were randomly assigned (random.org; Randomness and Integrity Services Ltd.) for the infusion of vehicle control, cultured or thawed MSCs (Supplementary Fig. S2). On Day 0 of the study, animals were anesthetized by intraperitoneal injection of a mixture of ketamine (120 mg/kg; Ketaset, Zoetis) and xylazine (6 mg/kg; Rompun, Bayer Inc.). The average weight of the animals was 19 g (range: 17–21 g). The cecum was isolated and ligated directly below the ileocecal valve followed by puncture of the cecum by an 18 G needle. Sham operation was performed by isolation of the cecum without ligation and puncture. Six hours post-CLP or sham operation, vehicle (Sham/vehicle, *n* = 6; CLP/vehicle *n* = 8), cultured (*n* = 12) or thawed (*n* = 11) MSCs (2.5 × 10^5^ cells, 100 µL total volume) were infused via the cannula inserted into the jugular vein as described previously^27^. Imipenem (Sigma) was given subcutaneously at 6 h after CLP. Twenty-four hours after the sham or CLP procedure, the animals were euthanized using standard lab protocol. Peritoneal lavage was harvested and plasma samples were also collected. Fresh tissue samples were unable to be collected from mice euthanized or found dead prior to the 24 hr pre-specified endpoint, and therefore these mice were not included in the analysis (incl. *n* = 4 for CLP/vehicle and *n* = 1 for CLP/thawed MSC). Cell infusion, animal wellness, and sample analyses were performed in a blinded fashion, with independent operators blinded for the group assignment.

### *In vivo* phagocytosis and bacterial clearance assays

Peritoneal cells were harvested by injecting 3 mL of phosphate-buffered saline (PBS; Gibco) intraperitoneally into mice using a 25 G needle. After gently massaging the abdomen for 1 min, 1 mL of lavage fluid was collected. Serial dilutions of the lavage fluid were plated on blood agar (trypt soya agar 5% BLD; Thermo Fisher Scientific) incubated at 37 °C overnight until colony forming units (CFU) were evident. The CFU were counted and the peritoneal bacterial load was calculated as total CFU counts. Additionally, peritoneal cells were centrifuged at 400 g for 5 min and washed with PBS supplemented with 3% fetal bovine serum (FBS; Gibco) and 0.2 mM ethylenediaminetetraacetic acid (EDTA; Invitrogen). Cells were incubated with pHrodo Bioparticles (Invitrogen) for 15 min at 37 °C, according to the manufacturer’s instruction, washed twice, stained with CD11b antibody (BD Biosciences) and analyzed using the Attune Acoustic Focusing cytometer (Invitrogen).

### Assessment of CLP-induced systemic inflammation

Plasma lactate levels were detected using Lactate Scout Test Strips (Sports Resource Group, Inc.). Plasma samples were analyzed by multiplex immunoassay using the Bio-Plex Pro II wash station (Bio-Rad) and Bio-Plex/Luminex 200 system (Bio-Rad) to detect systemic levels of CCL5, JE, a/KC, LIX, IL-10, and IL-1β, according to manufacturer’s protocol (Invitrogen).

### Statistical analysis

Statistical analysis was performed using GraphPad PrismV7.0 software (GraphPad Software, San Diego). Numerical data are presented as mean ± SEM unless otherwise stated. Multiple groups were analyzed by one-way ANOVA followed by Bonferroni’s multiple comparisons test unless otherwise stated. For the analysis of cytokine data, logarithmic transformation was performed to normalize the data distribution before conducting ANOVA. Statistical significance was set at *p* < 0.05.

### Data sharing

The data that support the findings of this study are available from the corresponding author upon reasonable request.

## Supplementary Information

Supplementary Information
