# Supplementary material for: Thawed Mesenchymal Stem Cell Product Shows Comparable Immunomodulatory Potency to Cultured Cells In Vitro and in Polymicrobial Septic Animals
Source: Sci Rep. 2019 Dec 2;9:18078. doi: 10.1038/s41598-019-54462-x (PMC6889371; doi:10.1038/s41598-019-54462-x)
Supplement: Supplementary file 1 — Supplementary Information [file 41598_2019_54462_MOESM1_ESM.docx]

**Online Data Supplement**

**Thawed Mesenchymal Stem Cell Product Shows Comparable Immunomodulatory Potency to Cultured Cells In Vitro and In Polymicrobial Septic Animals**

**Authors:**

Yuan Tan^1^, Mahmoud Salkhordeh^1^, Jia-Pey Wang^1^, Andrea McRae^1^, Luciana Souza-Moreira ^1^, Lauralyn McIntyre^2,3^, Duncan J. Stewart^1,3^, Shirley H.J. Mei^1*^

# Affiliations:

^1^ Regenerative Medicine Program, Ottawa Hospital Research Institute, Ottawa, Ontario, K1H 8L6, Canada

^2^ Clinical Epidemiology Program, Ottawa Hospital Research Institute, Ottawa, Ontario, K1H 8L6, Canada

^3^ Faculty of Medicine, University of Ottawa, Ottawa, Ontario, K1H 8M5, Canada

# * smei@ohri.ca

## Experimental Procedures

**MSC Isolation and Culture**

The human bone marrow aspirates were diluted with PBS (Gibco) and mixed with 3% acetic acid with methylene blue (STEMCELL Technologies) for counting of total nucleated cells via hemocytometer. Nucleated cells were plated on T-175 flasks (CELLBind) pre-coated with MSC Attachment Solution (Biological Industries) and containing complete MSC Nutristem xeno-free media (Nutristem XF basal medium [Biological Industries], Nutristem XF supplement mix [Biological Industries], gentamicin reagent solution [Gibco]). All cells were maintained at 37°C, 5% (v/v) CO_2_. Three days post-plating, a complete media change to MSC NutriStem XF medium was done. Every 3 days following, 30% of the serum free media was changed. The MSCs were passaged and transferred to HYPERFlasks (Corning) with 30% media changes occurring every 3–4 days. Upon 70–80% confluence, MSCs were harvested and cryopreserved at 2.5x10^6^ cells/mL in MSC freezing media (Biological Industries) in a CyroMED control-rate freezer (Thermo Scientific) with long-term storage in liquid nitrogen. Cryopreserved MSCs were thawed by immersing in a 37°C water bath for up to 2 minutes. All manufacturing occurred in a BioSpherix Xvivo isolation chamber system that included a complete modulator ISO 5 closed glove-box system housed in a dedicated ISO 8 clean room.

**EC permeability**

Endothelial cells were seeded in a transwell and treated with LPS for 6 hours. Cells were then co-cultured for 24 hours with MSCs that were seeded in the lower receiver wells. After co-culture, FITC-dextran was added to the top of the transwells. Permeability of the EC monolayer was determined by taking samples from the lower compartment to measure FITC-dextran levels. Relative permeability of each group was calculated by dividing the mean fluorescent intensity (MFI) of a given sample to the MFI of naïve ECs (without LPS treatment).

**Animals**

Animals were housed in conventional & breeding cages at five animal per cage and were fed Teklad Global 18% Protein Rodent Diet (Harlan Laboratories).

**
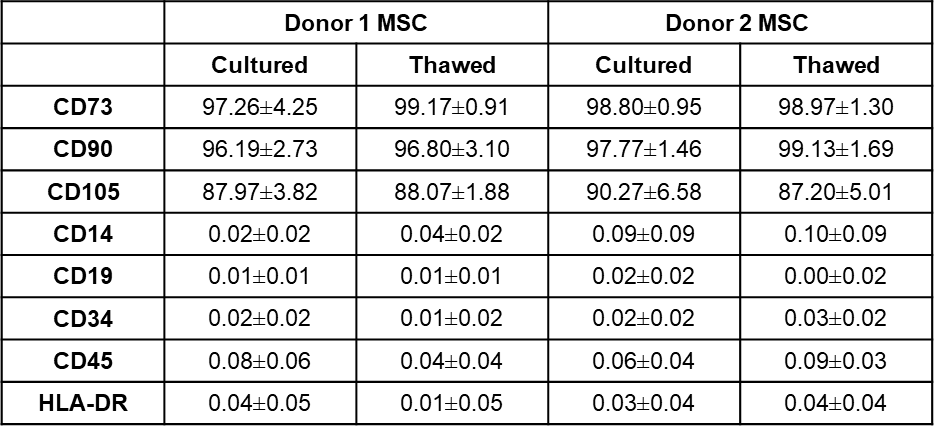
**

**Table S1.** MSC surface marker profiling at 4 hours after harvest (freshly cultured) or thaw (freshly thawed). *n* = 3 independent experiments for each donor, with data represent mean ± SEM.

**
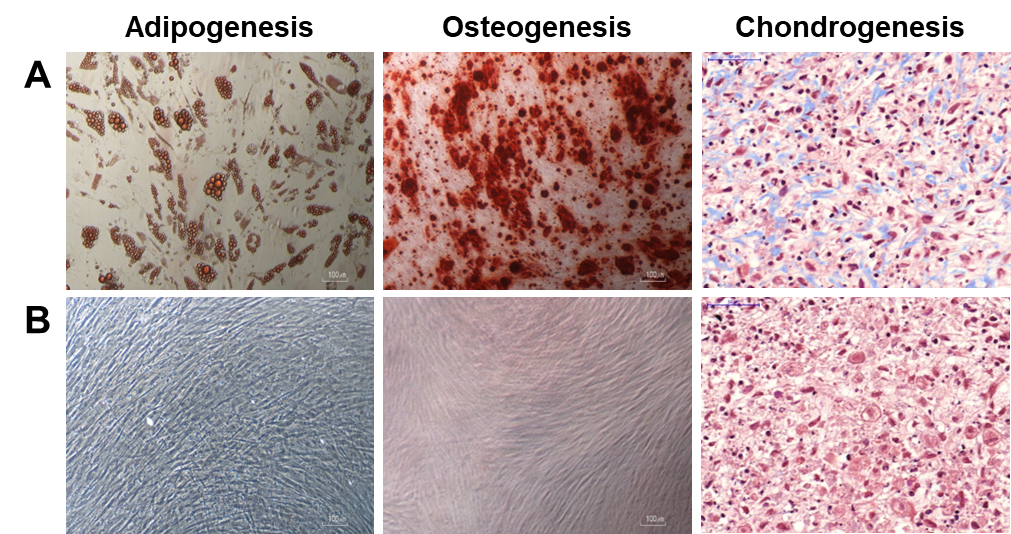
**

**Figure S1. Representative images of differentiated MSCs into tri-lineages.** MSCs were cultured with (A) or without (B) differentiation supplemented media for 14 days in 24-well plates (adipogenic supplemented cells), 21 days in 24-well plates (osteogenic supplemented cells), or in pellet form for 21 days in 15 mL conical tubes (for chondrogenic supplemented cells). Cells were fixed with 4% paraformaldehyde (for adipogenic, osteogenic and chondrogenic supplemented cells) as well as paraffin embedded for chondrogenic supplemented cells. Cells were stained with Oil Red O, Alizarin Red or Masson Trichrome for adipogenic, osteogenic or chondrogenic differentiation, respectively. Scale bar = 100 µm for adipogenesis and osteogenesis, and scale bar = 50 µm for chondrogenesis.

**Figure S2.** **Fluorescent intensity of FITC-dextran leakage after endothelial cells co-cultured with** **cultured and thawed MSCs post-LPS injury.** Group comparisons were analyzed by one-way ANOVA with Tukey's post hoc test. *****p*<0.0001, LPS-treated ECs versus non-treated group. ns = not significant between groups compared.

**
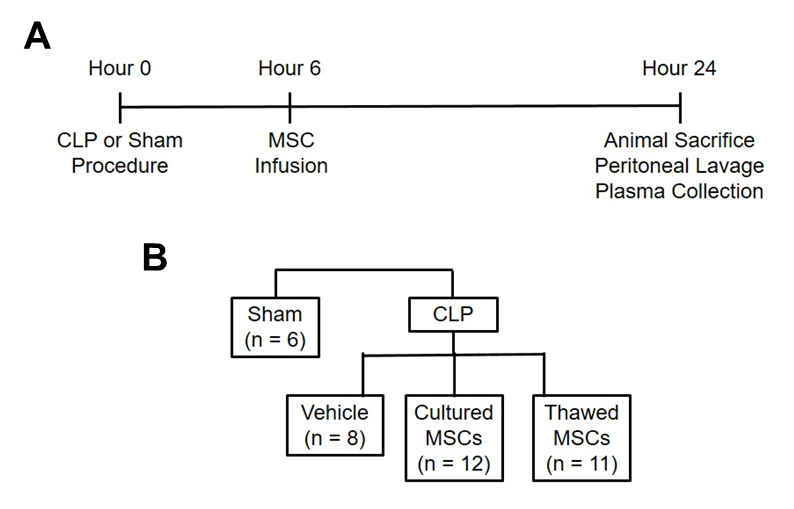
**

**Figure S3.** Experimental design of the cecal-ligation-puncture (CLP) murine model of polymicrobial sepsis (A) and flow chart of evaluated test groups (B).
